# A new Rothamsted long-term field experiment for the twenty-first century: principles and practice

**DOI:** 10.1007/s13593-023-00914-8

**Published:** 2023-08-24

**Authors:** Xiaoxi Li, Jonathan Storkey, Andrew Mead, Ian Shield, Ian Clark, Richard Ostler, Beth Roberts, Achim Dobermann

**Affiliations:** 1 Protecting Crops and the Environment, Rothamsted Research, West Common, Harpenden, , AL5 2JQ Hertfordshire UK; 2 Present Address: CSIRO Agriculture and Food, Canberra, ACT 2601 Australia; 3 Present Address: International Fertilizer Association, 49 avenue d’Iena, 75116 Paris, France

**Keywords:** Large-scale rotation experiment, Crop rotation, Tillage, Organic amendment, Crop protection

## Abstract

Agriculture faces potentially competing societal demands to produce food, fiber and fuel while reducing negative environmental impacts and delivering regulating, supporting and cultural ecosystem services. This necessitates a new generation of long-term agricultural field experiments designed to study the behavior of contrasting cropping systems in terms of multiple outcomes. We document the principles and practices of a new long-term experiment of this type at Rothamsted, established at two contrasting sites in 2017 and 2018, and report initial yield data at the crop and system level. The objective of the Large-Scale Rotation Experiment was to establish gradients of system properties and outcomes to improve our fundamental understanding of UK cropping systems. It is composed of four management factors—phased rotations, cultivation (conventional vs reduced tillage), nutrition (additional organic amendment vs standard mineral fertilization) and crop protection (conventional vs smart crop protection). These factors were combined in a balanced design resulting in 24 emergent cropping systems at each site and can be analyzed at the level of the system or component management factors. We observed interactions between management factors and with the environment on crop yields, justifying the systems level, multi-site approach. Reduced tillage resulted in lower wheat yields but the effect varied with rotation, previous-crop and site. Organic amendments significantly increased spring barley yield by 8% on average though the effect again varied with site. The plowed cropping systems tended to produce higher caloric yield overall than systems under reduced tillage. Additional response variables are being monitored to study synergies and trade-offs with outcomes other than yield at the cropping system level. The experiment has been established as a long-term resource for inter-disciplinary research. By documenting the design process, we aim to facilitate the adoption of similar approaches to system-scale agricultural experimentation to inform the transition to more sustainable cropping systems.

**Supplementary Information:**

The online version contains supplementary material available at 10.1007/s13593-023-00914-8.

## Introduction

### The evolution of agricultural field experiments and the need for a new approach

The history of systematic agricultural field experimentation can be traced back to the selection experiments of Gregor Mendel in the Czech Republic on the characteristics of pea plants in the 1850s and 1860s (Maat 2011). At around the same time, Sir John Bennet Lawes began a series of field experiments exploring the effect of varying combinations of nutrients on the yield of different crops on the Rothamsted Estate in the UK (Parolini 2015). If we define agricultural field experimentation as ‘the cultivation of crops with the primary aim of advancing knowledge’, the Rothamsted experiments can be viewed as the start of the modern era of agricultural science. Some of these original experiments still exist today as the longest running field experiments in the world, the oldest being the Broadbalk winter wheat experiment started in 1843 (Glendining et al. 2021).

Current approaches to agricultural field experimentation have been shaped further by the development of modern experimental design and statistics led primarily by Sir Ronald Fisher in the 1920s and other statisticians of that time, based on analyzing the old Rothamsted trials (Rao 1992). These advances largely determine the principles used in agricultural field experiments to this day, based on studying the effect of different levels of one or more ‘factors’ (e.g., rates of fertilizer or different varieties), on a single response variable (usually yield) incorporating the ideas of replication, spatial control (blocking) and randomization (Fisher 1925, 1935; Mead et al. 2012). These innovations in field experiment design have largely happened against the backdrop of the Green Revolution and the dominant policy agenda of increasing food production to feed a growing global population (Evenson and Gollin 2003). However, the negative environmental impacts of ‘production-oriented agriculture’ have increasingly come under scrutiny (Krebs et al. 1999), and the successes of the Green Revolution have come with unintended consequences including the loss of nutritional value, soil function, biodiversity and increasing chemical pollution (Pingali 2012). In response to these pressures, the agricultural policy agenda, especially in Europe, has been changing away from direct support for production to more environmentally sustainable farming. For instance, the UK is shifting to payment to farmers for production of public goods (Defra 2022), and the EU is reforming its common agricultural policy to align with the European Green Deal (EC 2021). Such policy changes necessitate a new approach to agricultural field experimentation to inform the design of cropping systems that better balance multiple sustainable development outcomes. These new ‘systems based’ experiments need to address two challenges that face conventional approaches to agricultural field experimentation.

Firstly, as opposed to having a focus on productivity as the main or only outcome, agricultural research now needs to capture the direct and indirect effects of management change on *multiple outcomes.* These outcomes may respond additively, synergistically, or antagonistically to a change in practice over different spatial and temporal scales. They include, among others, greenhouse gas (GHG) emissions, soil health, biodiversity and chemical pollution as well as crop yield and quality. Secondly, agricultural science is primarily an applied discipline with the aim of providing the evidence base to inform sustainable farming practice and government policy. Agricultural experiments typically have this applied goal in sight, providing solutions or optimization of management, e.g., parameterizing nitrogen response curves to optimize fertilizer use. However, there is unlikely to be one single solution to address the environmental issues facing agriculture while maintaining food production. Rather, there will be a complex array of alternative management trajectories that could move the system along a gradient of sustainability (assessed on multiple criteria) depending on the environmental and socio-economic context and starting point. For these reasons, it is impossible to arrive at the optimal solution through the conventional factorial treatment approach as empirically comparing all potential combinations of a wide range of alternative management practices in different environments is neither cost-effective nor practical.

In response to these challenges, several research groups around the world are adopting novel experimental approaches that make the cropping system the unit of study and compare the performance of alternative systems against multiple criteria (Adeux et al. 2019; Gathala et al. 2013; Hawes et al. 2018; Wolf et al. 2018). By including sufficient replication at the system level, the performance of different systems can be compared statistically based either on single response variables or multiple criteria (Davis et al. 2012). One approach to designing these experiments is to start with a set of environmental, agronomic or economic constraints or goals and to design systems that are predicted to meet these objectives (Debaeke et al. 2009). Management decisions are taken based on sets of ‘rules’ that differ between systems. In this case, the analysis is not based on a statistical comparison of the performance of the contrasting systems but on the probability of the desired objectives being met. Implicit in this approach is the opportunity to adapt cropping systems over time to further optimize systems against the defined constraints and goals (Colnenne-David and Doré 2015). An alternative approach is to establish experimental systems that differ as much as possible in terms of multiple properties that can be monitored in the long term to improve our understanding of system behavior to meet future challenges. This is more the philosophy of the Century Experiment in the USA that started in 1993 and is intended to run for 100 years (Wolf et al. 2018).

### Envisaging a new long-term experiment (LTE) at Rothamsted

**Fig. 1:**
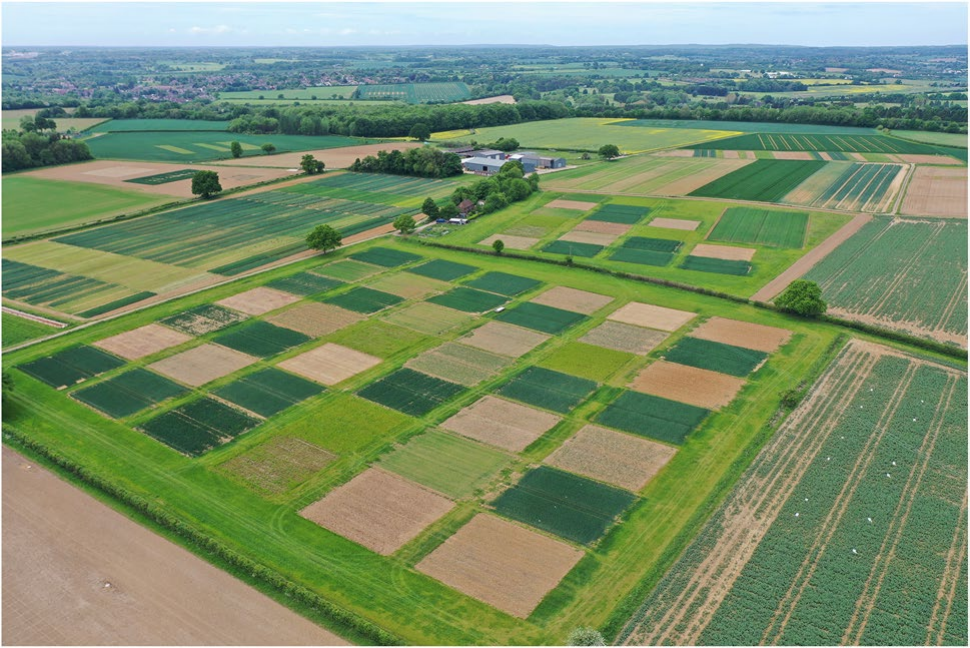
An aerial image taken on 17 May 2022 showing the Large-Scale Rotation Experiment (LSRE) that was established in 2018 at Rothamsted Research, Harpenden, Hertfordshire, UK. There are 60 square main plots with 18 in the field at the top right and 42 at the bottom of the image; an initial soil sampling survey confirmed the fields were similar and did not justify an additional blocking structure. The LSRE at the Harpenden site is adjacent to the Broadbalk Experiment that was started in 1843 (top left on the image with plots in long strips).

Building on the rich history of long-term agricultural experiments and statistics at Rothamsted Research, we report on the design and establishment of a new long-term, systems-based field experiment at Rothamsted (Fig. 1) and present initial results on productivity to illustrate our novel approach to the design and statistical analysis. We took the famous Broadbalk wheat experiment (Fig. 1)—the first experiment begun by Lawes in 1843 (Glendining et al. 2021)—as our starting point to ask the question: “what should a new Rothamsted LTE designed to address the 21^st^ Century challenges to UK agriculture look like?” and to establish some guiding principles for the design process (Table 1). The LTEs at Rothamsted are now used to address scientific questions that are often far removed from the original objectives of the experiments (Storkey et al. 2016). Rather, it is the divergence of the plots over decadal times scales and their contrasting responses to environmental variability and change that makes them a unique resource for agricultural scientists today. We chose, therefore, not to take the approach of designing the experiment to meet specific policy goals or environmental constraints (Colnenne-David and Doré 2015) that may change in the future. Rather, the overall objective of the new experiment was to establish an inter-disciplinary, long-term platform to improve our fundamental understanding of the behavior of UK cropping systems that is complementary to the existing Rothamsted LTEs. By establishing gradients of multiple state variables and outcomes across new experimental cropping systems that could be monitored over the long term, we intended the experiment to provide future evidence for:

1. Building a predictive framework of the impact of a change on multiple outcomes in de novo systems and so informing the design of more sustainable systems.
2. Identifying indicators of system properties that explain variance in outcomes and can be used to benchmark cropping systems and monitor change and progress toward sustainability.

**Table 1:** Design principles for a new long-term experiment compared to the Broadbalk wheat experiment, the world’s oldest continuous agricultural field experiment.

Broadbalk wheat experiment	New long-term experiment
Designed with a single agronomic outcome in mind—yield.	Establish trade-offs and synergies between multiple agronomic, environmental and economic outcomes.
Established before Fisher’s design principles and so lacks true replication and randomization.	Efficient design that retains sufficient statistical power to compare performance of contrasting systems against multiple outcomes and quantify contribution of individual management factors.
Originally focussed on a single factor (crop nutrition), though rotation and crop protection treatments added later.	Combine multiple factors to construct a set of emergent cropping systems.
Established at a single site (weather and soil), though now a well-characterized location.	Establish at more than one site with contrasting environments, to enable assessment of site-by-intervention interactions
Long thin plots (large edge effect), later split to incorporate additional treatments which however cannot be properly randomized.	Large square plots (smaller edge effects, more representative treatment impacts), providing more future flexibility in incorporating additional treatments, but a relatively uniform trial area required.
Constrained by historical legacy, but continues to provide useful insights through analysis of the impacts of variation in weather within a well-established system.	Flexibility to adapt treatments to new management or research questions.

The emphasis of this paper is on the principles and practices that were followed in the design and implementation of the experiment with reference to alternative statistical approaches to analyzing the data at different levels. Initial results are preliminary but are presented for one outcome (productivity) at both the crop and system level. This is to illustrate the power of our approach to the design and an example of statistical modeling of experiments of this type. We intend for the paper to be a resource for the agricultural science community as it adapts long-term experimental approaches to address modern day research needs.

## Materials and methods

### Design process

In 2015, a multi-disciplinary working group was set up to plan a new long-term experiment at Rothamsted. The group consisted of crop and soil scientists, microbiologists, ecologists, weed scientists, entomologists, pathologists and statisticians. Members of the Rothamsted Research Farm staff who would be responsible for the practical management of the experiment were also included in designing the experiment to capture important aspects of the practical agronomy that would need to be considered. It is important to emphasize at the outset that the new experiment was intended primarily as a resource for the agricultural research community. While the experimental cropping systems had to be agronomically realistic and relevant to current practice, they were not designed to identify the ‘optimal’ system that could be demonstrated to farmers. There was not, therefore, a wider stakeholder engagement at the planning stage, but this may have been beneficial for practical decision making around the management of the experiment.

The new experiment was centered on arable cropping systems that are adapted to the wheat growing areas of the UK. While the management factors are specific to this regional context, the design process we followed is generally applicable to other systems and environments. The design process followed the guiding principles established a priori (Table 1) and had four main stages:

1. **Identify outcomes:** the multiple societal demands on agricultural land in terms of agronomic, environmental and economic outcomes that needed to vary across the experimental cropping systems were identified.
2. **Define management factors:** potential ‘management factors’ that would be predicted to impact multiple outcomes identified in step 1 were reviewed. Factors were defined in terms of management rules within which specific treatments (e.g., sowing dates or fertilizer rates) are allowed to vary based on seasonality and the demands of the crops.
3. **Quantify statistical power:** alternative approaches to combining management factors in contrasting systems were explored that retained sufficient statistical power to both compare the performance of different cropping systems and to partition variance in outcomes to single management factors and interactions between them while accounting for practical constraints of space and cost.
4. **Identify measurement variables:** to inform both the baseline surveys of the experimental sites and ongoing sampling strategy a list of variables was compiled across the disciplines represented in the group that were predicted to respond to management factors and affect outcomes. Existing data from the site on variability in soil properties were also included in the discussion to inform any requirement for including a blocking structure in the design.

One of the limitations of the historical long-term experiments at Rothamsted is the lack of a formal statistical design. With this in mind, one of our guiding principles was to design the experiment in a way that retained sufficient statistical power to contrast systems and attribute variance in outcomes to component management factors (Table 1). This was done by taking the novel approach of using a factorial approach to combining management factors (described below) in a balanced design such that the plots represented emergent contrasting systems that could also be compared statistically. This contrasts with the usual approach to experiments of this type where systems are designed ‘top down’ against policy targets or environmental constraints.

### Identification of outcomes and management factors

The choice of management factors was guided by the aim of establishing contrasting cropping systems that differed in terms of their delivery against multiple outcomes. These outcomes were identified as being agronomic (productivity and nutritional quality), environmental (soil health, resource use efficiency, losses to the environment and biodiversity) and economic (inputs and farm profitability). Potential management factors were then chosen based on their expected impact on these outcomes; the intention being to select factors that would impact multiple outcomes to better understand trade-offs and synergies related to altered system behavior. Four management factors were selected defined by rules, discussed below: (1) three rotations (with either three, five or seven phases), (2) two intensities of cultivation, and two approaches to (3) crop protection and (4) crop nutrition. When combined, this resulted in 24 emergent cropping systems with unique combinations of rotation, cultivation, crop protection and organic amendments (Table S1).

**Fig. 2:**
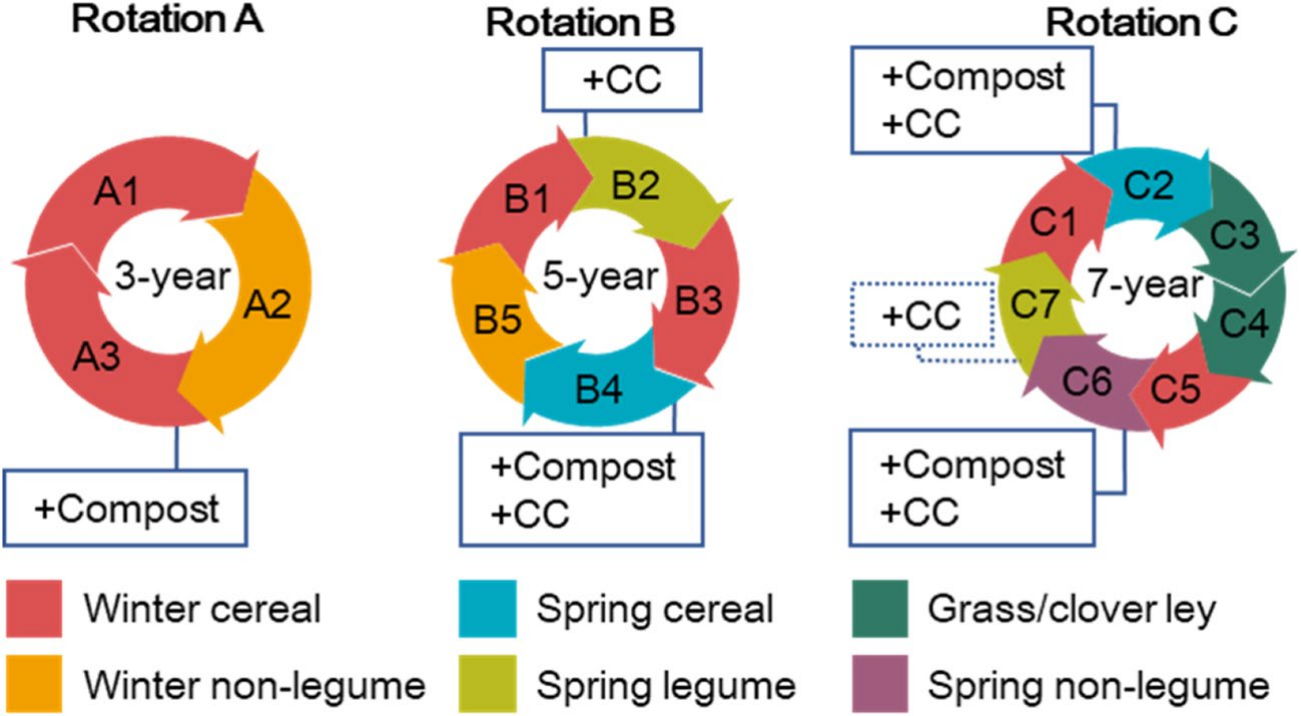
The rotation and nutrition factor of the new Large-Scale Rotation Experiment at Rothamsted Research. There are three crop rotations of 3, 5 and 7 years (indicated by A, B and C, respectively) and two levels of nutrition, standard fertilization and amendment with additional organic materials in terms of cover crops (CC) and/or compost. All 15 phases are present every year at each site, i.e., design is fully phased. The same crop is used for each phase at both sites except for C6 where sugar beet was sown at Brooms Barn while linseed was sown at Harpenden. Organic amendments are added at the subplot level to selected phases. CC is generally grown preceding a spring crop, except that preceding C7 at Brooms Barn due to late harvest of sugar beet (C6). To be noted, CC is a non-legume mix in rotation B but a mix of legumes and non-legumes in rotation C. For the cultivation and crop protection factor, see Fig. 3.

Three crop rotations (3-year, 5-year and 7-year) were included in the experimental design (Fig. 2); these represented a gradient of crop functional diversity while being agronomically realistic. Following the principle of incorporating flexibility into the experimental design, the phases in each rotation are defined at the level of crop functional group, affording the opportunity to substitute crop species between years (Fig. 2). Crops included in the rotations are winter wheat [*Triticum aestivum* L.] (W), winter oilseed rape [*Brassica napus* L.] (OSR), spring barley [*Hordeum vulgare* L.] (SBa), spring field beans [*Vicia faba* L.] (SBe), soybean [*Glycine max* (L.) Merr.] (Soy, failed and replaced by SBe since 2020), sugar beet [*Beta vulgaris* L. ssp. *vulgaris*] (only at Brooms Barn) and linseed [*Linum usitatissimum* L.] (only at Harpenden). A 2-year grass/clover ley (GC) was included in the 7-year rotation. Sowing and harvest dates of the crops along with information on typical nitrogen fertilizer rates are provided in Fig. S1. Where year is referred to throughout the text, this means the year of harvest unless stated otherwise. To allow the confounding effects of season to be fully captured in analyses of the data, the experiment followed a ‘fully phased’ approach—every phase of each rotation being present every year. A total of 15 phases thus formed a nested structure within the rotation factor.

Each crop phase was either cultivated using conventional inversion tillage or reduced tillage. For conventional tillage, moldboard plowing was performed prior to seedbed preparation and drilling using an Accord 3m combination drill. For reduced tillage, seeds were drilled directly into the stubble of the preceding crop using a Weaving 3m GD direct drill at Brooms Barn and a Simtech 3m T-Sem 300 drill at Harpenden, minimizing any additional soil disturbance. While the ideal was ‘zero-till’ on these subplots, some sub-soiling or minimal surface disturbance was permitted if the plots needed to be leveled.

Half of the main plots at each site were designated as ‘smart crop protection’ (SCP). These were managed in a way to reduce inputs of pesticides and included the following practices: growing cultivar mixtures, spraying to pest and disease thresholds, delaying drilling to control weeds and companion cropping. The SCP treatment can be envisaged as employing some of the principles of Integrated Pest Management (Barzman et al. 2015) that are relevant to specific crops. Other principles, for example crop diversification, are captured in the wider design of the experiment. The other half of the plots were managed following conventional crop protection approaches.

The nutrition factor comprised either a standard mineral fertilization approach following local recommendations and soil testing or this standard approach but with the addition of organic amendments, applied either as a living mulch (cover crop, CC) and/or a dead mulch (green compost) (Fig. 2). Cover crops were grown over winter prior to all spring crops, and compost was added to selected phases in September, both on the amended subplots. The cover crop was a legume and non-legume mix used in the 7-year rotation but only a mix of non-legumes in the 5-year rotation (Fig. 2) to establish contrasting diversity of mixes. The compost was produced from garden plant residues by a commercial company (for details of the compost see Supplementary Materials). This approach was intended to achieve a large contrast in some properties (e.g., soil organic carbon) between the amended and standard treatments in the long term.

### Site description

The resulting experiment, called the Large-Scale Rotation Experiment (LSRE), was established at two sites in south-eastern England, Brooms Barn, Suffolk (52°15′46.60″N, 0°33′54.79″E) in autumn 2017 and Harpenden, Hertfordshire (51°48′40.24″N, 0°22′18.65″W) in autumn 2018, with the field at Harpenden being adjacent to the Broadbalk experiment. The experimental sites have contrasting soil types, classified using the Soil Survey of England and Wales (Clayden and Hollis 1984): Brooms Barn is a Moulton series sandy loam (Clay 14.8%, Silt 20.7%, Sand 64.5%), and Harpenden is a Batscombe series silty clay loam (Clay 24.9%, Silt 51.5%, Sand 23.6%). The contrasting sites were included to quantify the importance of environmental factors in determining the response of different outcomes to the management factors. We would expect the legacy of previous field management on soil properties (determining the starting point for change) to also effect outcomes (the fields had previously been used for short term experiments); to account for this and to test for any spatial heterogeneity in soil properties a detailed baseline sampling campaign was done in the stubble of a preparatory crop of winter oats at each site (described in Supplementary Materials). Selected additional soil properties from the baseline sampling are shown in Table 2. The long-term (1991–2020) average annual precipitation and air temperature were 643.6 mm and 10.5℃ at Brooms Barn, and 763.5 mm and 10.2℃ at Harpenden (Supplementary Table S2 and S3).

**Table 2:** Baseline soil properties measured at the two experimental sites.

	Unit	Brooms Barn			Harpenden		
		0–23 cm	23–60 cm	60–100 cm	0–23 cm	23–60 cm	60–100 cm
Soil organic carbon	%	0.89	0.43	0.25	1.66	0.93	0.39
Total N	g kg^−1^	0.92	0.49	0.30	1.56	1.01	0.58
Olsen P	mg kg^−1^	23.6	10.1	5.8	29.8	12.7	3.6
Exchangeable K	mg kg^−1^	89.6	46.4	55.8	188.0	161.3	120.9
Exchangeable Mg	mg kg^−1^	37.0	32.7	47.8	82.0	69.1	50.0
Exchangeable Mn	mg kg^−1^	4.9	3.4	0.5	10.3	2.8	0.7
Exchangeable Ca	mg kg^−1^	1292.4	1552.0	2403.8	2398.0	3054.0	4123.8
pH	-	7.13	7.52	7.86	7.30	7.69	7.90

The Brooms Barn site has traditionally been used for sugar beet research, and beet was included as phase C6 in the 7-year rotation; a single plowing event is unavoidable following the sugar beet harvest meaning plots in the reduced tillage treatment are inverted every seventh year at this site. Additionally, it is usually harvested late which leaves little opportunity for a cover crop; therefore, a cover crop preceded the C7 at Harpenden but not at Brooms Barn (Fig. 2). At Harpenden, linseed is grown at C6 in the rotation meaning conventional plowing is never used on the reduced tillage plots.

### Statistical design

**Fig. 3:**
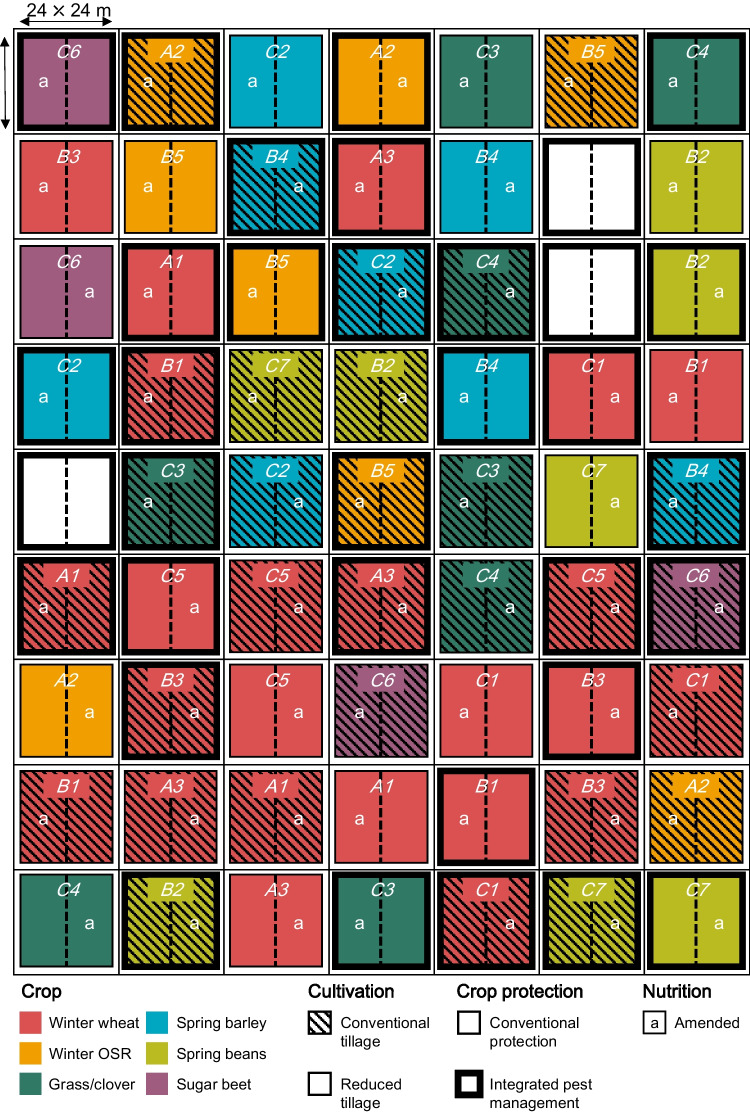
Plot layout map of the Large-Scale Rotation Experiment illustrated with crops harvested at Brooms Barn site in 2021. All phases (15) of the 3-, 5- and 7-year rotations (indicated by A, B and C on the map, respectively) are sown every year and established either using conventional tillage (plots with stripes) or reduced tillage (plots without stripes). Fully randomized main plots are split and organic amendments added to one half (as cover crops and/or compost), which is indicated by ‘a’ on the map. The amendment is intended to be restricted to only one side of the plot to enlarge the contrast of the soil organic carbon content in the long run. Additionally, they were only added to selected phases rather than to all plots in each year (for details see Materials and methods, and Fig. 2). The plots with thin border lines on the map have conventional crop protection management, whereas those with thick border lines are managed using smart crop protection principles for weed, pest and disease control. Therefore, each subplot is a unique system subject to a combination of management factors of phase, cultivation, nutrition and crop protection. At Brooms Barn, the regular arrangement of plots in the field meant there were three ‘blank’ plots. OSR indicates oilseed rape.

The experiment at each site was a completely randomized factorial split-plot design. Three of the four factors—phase (nested within rotation), cultivation and crop protection—were fully crossed and allocated at the main-plot level, while the nutrition factor was applied randomly at the subplot level, i.e., each main-plot was split into two halves. The experimental fields were both relatively homogenous, and an additional blocking structure was not justified. The combined management factors resulted in a total of 60 main-plots (24 × 24 m each) and 120 subplots at each site (Fig. 1 and 3). An important outcome of this design process was that once all the factors were combined, there is no true spatial replication within a site for the highest order interaction. However, the design incorporates ‘hidden replication’ and the power to analyze lower order interactions (as illustrated below). The fact that the experiment is also ‘fully phased’ also means that data across all the phases of a system can be integrated within a given year (space for time substitution) to run models at the level of the system. Short runs of contiguous years can then be used as replicates, assuming that they share a similar legacy effect. In later years, when individual plots have completed one or multiple cycles of the rotations, spatial replicates of the different system will exist at each site (albeit unbalanced).

**Fig. 4:**
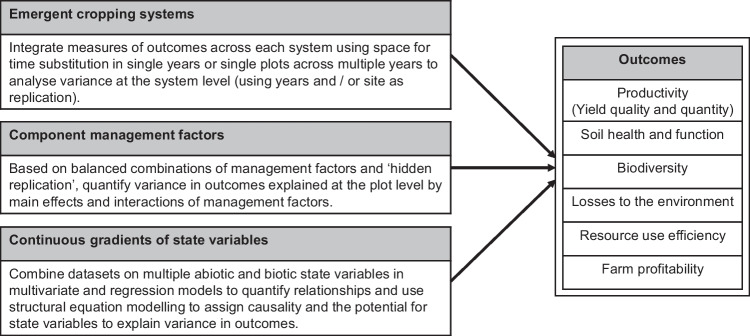
Alternative approaches to modeling outcomes on the LSRE at different levels of organization.

**Fig. 5:**
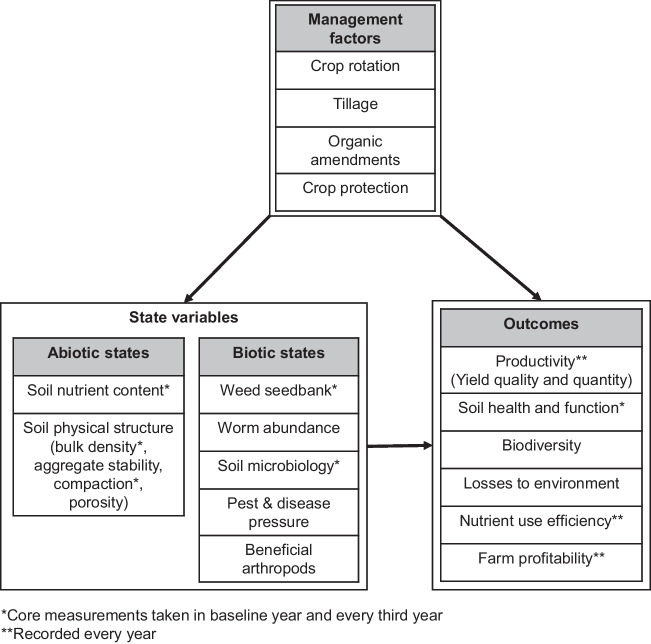
Summary of management factors and variables measured in the LSRE. Yield and yield quality (protein and oil content) are measured in all years; a core set of additional measurements were taken in the baseline year and are repeated every third year at each site (see Supplementary Materials). An alternative approach would be to sample every third, fifth and seventh year to coincide with completed rotations that will be considered in the future. These are supplemented with additional data on other variables taken as part of associated projects using the experiment as a platform.

The LSRE was designed with three main contrasting approaches to data analysis in mind (Fig. 4). Firstly, the combination of rotation × cultivation × crop protection × nutrition factors results in 24 emergent cropping systems (Table S1). As all phases of the rotations are present every year, data can be integrated across plots using space for time substitution and year and site used as replicates to allow the performance of the systems to be compared statistically in terms of outcomes. Secondly, the factorial treatment structure associated with the four management factors also allows the assessment of the main effects and low-order interactions on outcomes within an analysis of variance (ANOVA) framework. Finally, the co-variance of state and outcome variables in response to the treatments can be analyzed using multivariate analysis and multi-variable modeling including structural equation models (Garland et al. 2021) (Fig. 5). More detailed statistical considerations when designing the LSRE can be found in Supplementary Materials.

### Analysis of productivity

For the purposes of this paper, we illustrate the first and second approach to analyzing the LSRE, described above. We analyze data on productivity to demonstrate the power of combining management factors in a balance design to create contrasting cropping systems using linear mixed effects models. As state variables respond over different timescales and datasets currently vary in completeness, the multivariate analyses across data types will be reported in future publications. Yields are recorded on all subplots in all years at both sites from two yield strips running the length of each subplot and a width of 1.5 and 2 m (an area of 36 and 48 m^2^) for each strip using a plot combine at Brooms Barn and Harpenden, respectively. A subsample of the grain is oven-dried at 105℃ overnight to obtain the actual moisture content. The final yield is expressed as weight at 15% moisture content for wheat, barley and beans and 9% for OSR.

To exemplify a system-level analysis, yields of individual crops were converted to calories for the 3-year and 5-year rotations in 2020 and 2021. As the crop grown in phase C6 differs between the Harpenden and Brooms Barn sites, and there are alternative ways of calculating calorific yield from grass leys depending on how it is managed, the 7-year rotation was not included in this analysis. The human-available calories of the four crops—wheat, OSR, barley and beans—were calculated using conversion factors from the USDA Nutrient Database (USDA Food Data 2019, compiled in Table S1 in Driscoll et al. 2022). Annual system calorific yield was calculated as the average over all phases of each system that were present in the same year at each site, and year was taken as replication (*n*=2), although, as more years of data become available, it can be averaged over the years for each system when data are accumulated in a longer period taking phases as repetition. An initial analysis using a linear mixed model and including system (i.e., the combination of the four management factors), site and their interaction as fixed effect terms showed no significant site effect, and then the model was updated to include only system as fixed effect. The random effect was included in the nested form: *random=~1 | Site/Year*.

We then analyzed the experiment at the plot level using the yields of three focal crops, winter wheat, spring barley and spring beans as response variables for which there were complete datasets in 2020 and 2021 from both sites. Because of OSR failures on some plots, there were insufficient data to include them in the plot-level analysis, but OSR was included in the calculation of average calorific yields (using zero when no crop was present) in the system level analysis. Firstly, a global linear mixed model was built including crop and site as fixed effects together with the four management factors. All four-way and simpler interaction terms were included in the fixed effects. Random effects were included in the nested form: *random=~1 | Site/Mainplot/Subplot/Year*. This allows the observations in different years to provide replication of the different treatment combinations, though clearly different main plots contribute data for each crop in the different years. Based on this initial multi-crop analysis, separate linear mixed models were also fitted to subsets of data for individual crops but retaining site in the fixed effects model. The fixed effects included for each model were rotation, cultivation, nutrition and crop protection, site (and previous-crop for wheat only) and all four-way and simpler interactions. As rotation and previous-crop were not fully crossed for wheat (Fig. 2), a new variable (*Rt.Pre*) was created to represent the seven combinations of the two factors. As all spring barley was only preceded by wheat, previous-crop was not included in the model for barley. Previous-crop was partially confounded with site for beans as the previous-crops were wheat (at both sites), sugar beet (only at Brooms Barn) and linseed (only at Harpenden). A new variable (*St.Rt*) was created to represent the site and rotation combinations in the model for beans. The nested random effect was the same as for the global model described above.

Details of all models used can be found in Supplementary Materials. All linear mixed models were fitted using the function lme from the R package nlme (Pinheiro and Bates 2000). All statistical analyses were performed using R v4.0.2 (R Core Team 2020).

## Results

### System level calorific yield

**Fig. 6:**
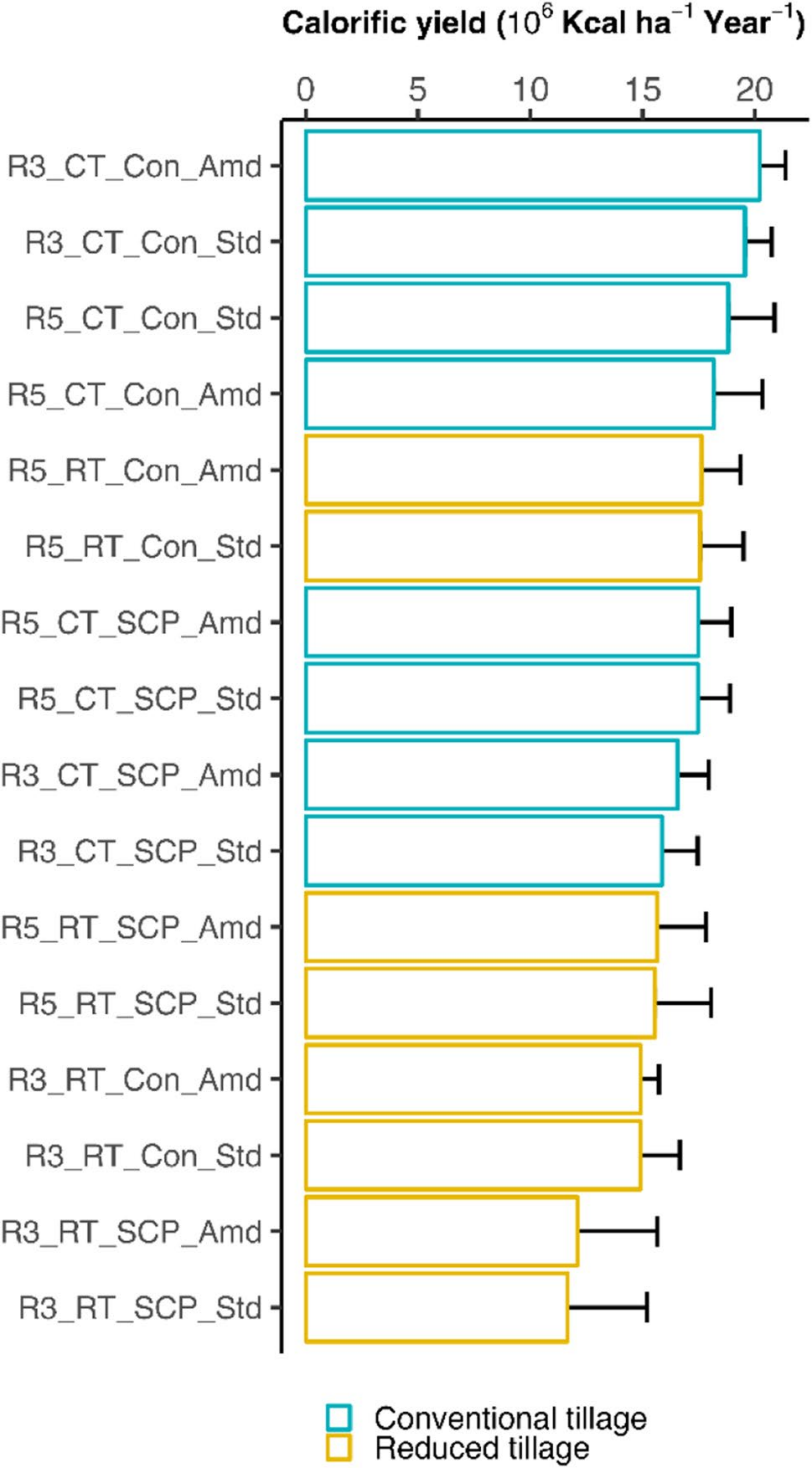
Calorific yield of different cropping systems. Data included were from the 3-year (R3) and 5-year (R5) rotation at both sites in 2020–2021. CT conventional tillage, RT reduced tillage, Con conventional crop protection, SCP smart crop protection, Amd standard fertilization plus amendment with additional organic materials, Std standard fertilization.

There was a significant cropping system effect on the annual system calorific yield (*p*<0.001), Fig. 6. The amended and standard plots for a given rotation × cultivation × crop protection system were always found together suggesting this factor has yet to play a big role in differentiating system productivity. However, in only one case was the standard system (no organic amendment) more productive than the amended plot. There was an indication that reduced tillage and smart crop protection had a detrimental effect on productivity; the most productive systems of the 3- and 5-year rotations were all plowed with conventional crop protection. It is likely that the frequency of OSR crop failures was important in determining the ranking of systems; as OSR represents a higher proportion of total production in the 3-year system, any failures will have a disproportionate effect in those systems (the four least productive systems were all in the 3-year rotation).

### Crop-level grain yield of LSRE (2020–2021)

There were significant interactions (*p*=0.021) between rotation, crop, cultivation and site on the yield of wheat, barley and beans of the LSRE in 2020–2021 when all data were analyzed together with a global linear mixed model (Table 3). The difference in cultivation effects on yield varied between sites, crops and the rotation systems in which the crops were grown. This was expected based on contrasting soil types at the two sites and justified the establishment of the LSRE at both sites. During the study period, the plots under reduced tillage tended to be consistently under-yielding compared with those under conventional tillage though effect sizes varied by crop, site and year (Table S4).

**Table 3:** Summary table for the global analysis on all yield data (*n* = 316) of the three crops (wheat, barley and beans) across the two sites during 2020–2021 using a linear mixed model.

	df	*p* value
Cultivation	1	0.199
Protection	1	0.199
Nutrition	1	0.506
Site	1	0.662
**Cr.Rt**	**6**	**<0.001**
Cultivation×Protection	1	0.514
Cultivation×Nutrition	1	0.233
Cultivation×Site	1	0.885
Protection×Nutrition	1	0.930
Protection×Site	1	0.354
Nutrition×Site	1	0.859
Cultivation×Cr.Rt	6	0.761
Protection×Cr.Rt	6	0.705
Nutrition×Cr.Rt	6	0.948
Site×Cr.Rt	6	0.703
Cultivation×Protection×Nutrition	1	0.393
Cultivation×Protection×Site	1	0.187
Cultivation×Nutrition×Site	1	0.339
Protection×Nutrition×Site	1	0.619
Cultivation×Protection×Cr.Rt	6	0.811
Cultivation×Nutrition×Cr.Rt	6	0.964
**Cultivation**×**Site**×**Cr.Rt**	**6**	**0.021**
Protection×Nutrition×Cr.Rt	6	0.975
Protection×Site×Cr.Rt	6	0.873
Nutrition×Site×Cr.Rt	6	0.994
Cultivation×Protection×Nutrition×Site	1	0.378

As crop and rotation were partially crossed (i.e., not all combinations present), a new variable, *Cr.Rt*, was created to represent combinations of the two factors in the model. The random effect was specified as a nested structure, *random = ~1 | Site/Mainplot/Subplot/Year*. *p* values were derived from Wald Chi-square tests

Significant effects (*p<0.05*) are indicated in bold

**Fig. 7:**
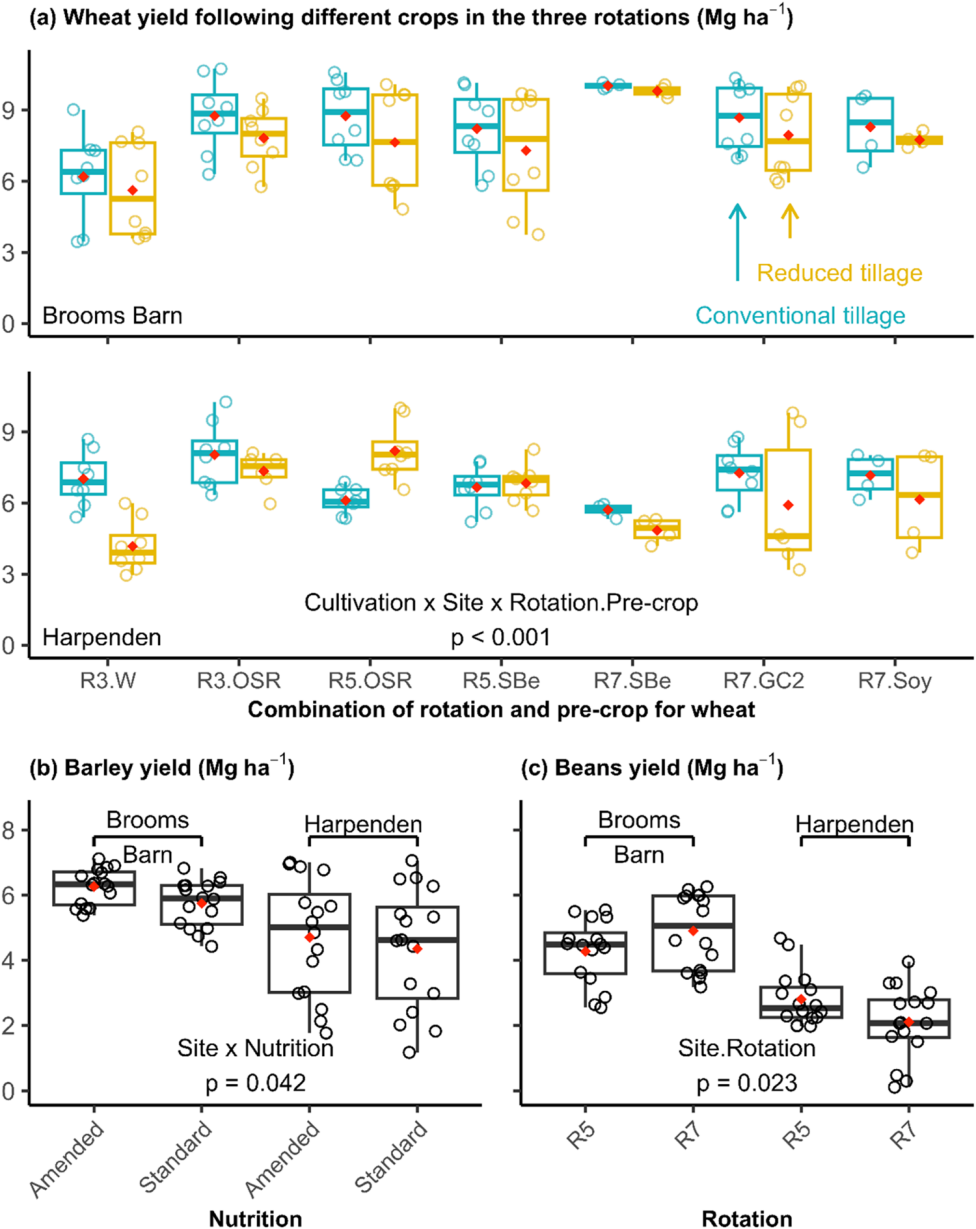
Yield of wheat (**a**), barley (**b**) and beans (**c**) at both sites in 2020–2021. Only the significant interaction of the highest order is displayed on the graph (For details see Table 4). The x-axis displays the combination of rotation and previous-crop (Rotation.Pre-crop) for wheat on (**a**), the nutrition factor (additional organic amendment vs standard fertilization) for barley on (**b**) and the rotation factor for beans on (**c**). R3, R5 and R7 indicated the 3-, 5- and 7-year rotations, respectively. W wheat, OSR winter oil seed rape, SBe spring beans, GC2 the 2^nd^ year grass/clover ley, Soy soybean. Mean values are indicated by diamond points. The lower and upper hinges correspond to the 25^th^ and 75^th^ percentiles, and the whiskers extend from the hinge to the largest or smallest value but no further than 1.5 times of the inter-quartile range from the hinge.

When yield was analyzed separately for individual crops during 2020 and 2021, the different management factors affected the yield differently for the three crops (Table 4, Fig. 7). There were three significant complex interaction effects on wheat yield, i.e., between cultivation, site, previous-crop and rotation (*p*<0.001), between cultivation, nutrition and site (*p*=0.049) and between cultivation and nutrition (*p*<0.001). Organic amendments significantly improved spring barley yields by 8% on average and the effect size varied by site (*p*=0.042 for nutrition × site, Table 4, Fig 7b). Moreover, the three-way interaction of protection × nutrition × site also showed a marginally significant effect on barley yield (*p*=0.051). It is worth noting that smart crop protection was only applied to winter crops, not to spring crops, so any differences are indicative of a legacy effect from the preceding crop. A greater yield was observed for spring beans at Brooms Barn than at Harpenden by 79% on average (56% and 103% in 2020 and 2021, respectively), and the site effect varied with rotation (*p*=0.023, Table 4, Fig 7c). These results indicated that different crops responded in different ways to management factors and to the site environments, demonstrating the need for experiments like the LSRE, that consider multiple factors at more than one site.

**Table 4:** *p* values of the fixed effects included in the linear mixed models for each crop.

Wheat (188)			Barley (64)			Beans (64)		
Fixed effect	df	*p* value	Fixed effect	df	*p* value	Fixed effect	df	*p* value
Ct	1	0.180	Rt	1	0.903	Ct	1	0.372
Pr	1	0.256	Ct	1	0.981	Pr	1	0.904
Nu	1	0.116	Pr	1	0.842	Nu	1	0.322
St	1	0.951	**Nu**	**1**	**0.011**	**St.Rt**	**3**	**0.023**
Rt.Pre	6	0.976	St	1	0.585	Ct×Pr	1	0.919
Ct×Pr	1	0.267	Ct×Rt	1	0.881	Ct×Nu	1	0.698
**Ct×Nu**	**1**	**<0.001**	Pr×Rt	1	0.928	Pr×Nu	1	0.969
Ct×St	1	0.219	Nu×Rt	1	0.824	Ct×Pr×Nu	1	0.920
Pr×Nu	1	0.810	St×Rt	1	0.715	Ct×St.Rt	3	0.947
Pr×St	1	0.453	Ct×Pr	1	0.814	Pr×St.Rt	3	0.667
Nu×St	1	0.724	Ct×Nu	1	0.875	Nu×St.Rt	3	0.278
Ct×Rt.Pre	6	0.997	Ct×St	1	0.376	Ct×Pr×St.Rt	3	0.943
Pr×Rt.Pre	6	0.983	Pr×Nu	1	0.139	Ct×Nu×St.Rt	3	0.869
Nu×Rt.Pre	6	0.649	Pr×St	1	0.870	Pr×Nu×St.Rt	3	0.090
St×Rt.Pre	6	0.055	**Nu**×**St**	**1**	**0.042**			
Ct×Pr×Nu	1	0.091	Ct×Pr×Nu	1	0.842			
Ct×Pr×St	1	0.233	Ct×Pr×St	1	0.830			
**Ct×Nu×St**	**1**	**0.049**	Ct×Nu×St	1	0.271			
Pr×Nu×St	1	0.475	Pr×Nu×St	1	0.051			
Ct×Pr×Rt.Pre	6	0.832	Ct×Pr×Rt	1	0.918			
Ct×Nu×Rt.Pre	6	0.175	Ct×Nu×Rt	1	0.734			
**Ct**×**St**×**Rt.Pre**	**6**	**<0.001**	Ct×St×Rt	1	0.941			
Pr×Nu×Rt.Pre	6	0.693	Pr×Nu×Rt	1	0.795			
Pr×St×Rt.Pre	6	0.613	Pr×St×Rt	1	0.466			
Nu×St×Rt.Pre	6	0.849	Nu×St×Rt	1	0.327			
Ct×Pr×Nu×St	1	0.094	Ct×Pr×Nu×Rt	1	0.391			
			Ct×Pr×St×Rt	1	0.795			
			Ct×Nu×St×Rt	1	0.684			
			Pr×Nu×St×Rt	1	0.080			
			Ct×Pr×Nu×St	1	0.544			

Data were from both sites in 2020 and 2021, and the number of observations were in parentheses following the respective crop name. Due to the partially crossed nature of variables, a new variable, *Rt.Pre*, was created to represent all available treatment combinations of rotation and previous-crop for wheat, and *St.Rt* for all combinations of site and rotation for beans. *Ct* cultivation (conventional tillage vs reduced tillage), *Pr* crop protection (conventional vs smart crop protection), *Nu* nutrition (standard fertilization vs additional organic amendment), *St* site, *Pre* crop preceding wheat (previous-crop), *Rt* crop rotation (3-, 5- or 7-year). *p* values were derived from Wald Chi-square tests. Significant terms (*p*<0.05) are highlighted in bold

## Discussion

While there is a consensus around the need for new, ambitious agricultural experiments that compare the behavior of whole cropping systems as opposed to individual treatment effects, there are alternative approaches to their design which reflect different philosophies and objectives. We would argue that these differences are largely related to the timescale over which an experiment is intended to run and became apparent during the process of designing the LSRE. Where short to medium-term objectives can be clearly identified (for example to reduce pesticide inputs or achieve net-zero carbon emissions) it is apposite to design experimental cropping systems to meet those objectives that can then be compared based on whether or not they succeed (Debaeke et al. 2009). Trade-offs with other performance criteria can then also be quantified. In this sense, the systems are designed ‘top-down’ and are bespoke to the defined objectives or environmental constraints. They can also be adapted within the lifetime of the experiment following a prototyping methodology (Colnenne-David and Doré 2015). In contrast, if the intention is to establish a long-term experimental platform, it may not be possible to anticipate the policy environment or research priorities of the future. The objective then becomes establishing systems that differ in terms of multiple system properties that can be monitored over time to improve our understanding of the behavior of the system to meet future challenges.

The design process of the LSRE reflected its long-term nature and the fact that it was not being established primarily as a response to the contemporary needs of policy or practitioners but as an enduring resource for the agricultural research community. In this sense, it is deliberately ‘open-ended’ without a specific destination in mind, providing an inter-disciplinary platform that is complementary to the existing suite of LTEs at Rothamsted. The choice of management factors was not, therefore, determined by hypotheses but rather by a discussion across disciplines as to the management ‘levers’ that would impact as many system properties as possible that will ultimately determine variability in outcomes. This has created a challenge in terms of communicating the experiment to policy makers and farmers who are interested in short-term solutions. Because the experimental systems are not designed to meet specific goals, it is not possible to identify an optimal system (that will depend on assigning relative value to outcomes). However, by presenting data on outcomes as, for example, radar plots for contrasting systems inherent trade-offs and synergies can be identified and discussed in the context of designing future cropping systems that meet alternative criteria. We have also found the LSRE to be a useful platform for discussing alternative approaches to agriculture—variously described as sustainable intensification (Pretty et al. 2018), agroecological (Wezel et al. 2020), regenerative (Giller et al. 2021) or conservation agriculture (Giller et al. 2015) with different stakeholders. The LSRE include all the ingredients included in these more sustainable paradigms (crop diversification, reduced tillage, increased organic inputs, reduced pesticides, increased green cover) in different combinations and has been useful for demonstrating the importance of understanding complex interactions when designing sustainable cropping systems (see discussion, below, on conservation agriculture).

A second challenge that we faced in the design process was the ambition to incorporate sufficient statistical power in the design to both be able to compare systems and to attribute variance in outcomes to individual and combined management factors. The decision was made early on to ‘build’ emergent cropping systems ‘bottom-up’ using balanced combinations of management factors. However, designing a conventional replicated block experiment that captured all the management factors we wanted to include while controlling for required space and costs was not possible. We believe the solution of employing ‘hidden replication’ in the design to be an effective compromise, retaining sufficient power to analyze the response of an outcome to main effects of the management factors or lower order interactions. However, in the initial years of the experiment, before all the plots have completed a full rotation, this means there is no spatial replication of each of the 24 unique cropping system within each site. This highlights a further challenge to establishing long-term experiments of this type. Realizing the scientific ambition of the LSRE will rely on the collection of contrasting data types from multiple disciplines, systematically accrued over time with the value of the experiment increasing with its age. In addition, the impact of the treatments on some state variables (e.g., soil organic carbon) will take many years to manifest, and experiments of this type, by their very nature, require a long-term commitment (Johnston and Poulton 2018; MacLaren et al. 2022). However, there is also the imperative to demonstrate the value of the experiment in the shorter term. Because the LSRE is fully phased, there is the opportunity to integrate data across all the plots in a system in a given year and use site and/or year as replicates. In this paper, we have demonstrated this approach for calorific yield, but similar calculations could be done for GHG budgets, nutrient use efficiency or profitability, either based on empirical data or simulation models parameterized on the different systems. As the experiment ages, care will need to be taken as years will increasingly vary in terms of the length of any legacy effect but given a sufficient temporal gradient could then be analyzed in terms of contrasting trends between experimental systems.

Although limited to the early transition years of the experiment, the analysis of productivity yielded some interesting results. In the global analysis of yield responses, several significant interaction effects on crop yield, i.e., between rotation, crop, cultivation and site, were observed for all three crops included in the analysis (Table 4). The 2020 season was wetter than the long-term average (1991–2020) during the study period (Tables S1 and S2) and the higher than usual precipitation coincided with lower winter wheat yields, probably due to poor establishment (Table S4). These inter-annual weather effects differed between sites (with the Harpenden site being proportionally wetter), which also had contrasting soil properties, explaining the interaction of cultivation effects with site. These results are also partly explained by the frequency of crop failures in the contrasting treatments; where winter wheat crop failed it was redrilled usually with a spring wheat variety. This was done with reference to the treatment structure; for example, all winter wheats in the conventional tillage plots at Harpenden in 2020 were redrilled, meaning that any effects of re-drilling could be considered part of the treatment effects on yields. In a global meta-analysis on the effect of reduced tillage on crop yield, Pittelkow et al. (2015) discussed that yield reduction associated with reduced tillage may be attributable to poor establishment and waterlogging.

Effects of reduced tillage on crop yield vary with multiple crop and environmental factors, e.g., crop type, water stress, duration of reduced tillage and soil conditions (Pittelkow et al. 2015; Samson et al. 2019). We observed a general trend of lower yields in the reduced tillage than the conventional tillage systems, though only statistically significant for wheat and not for beans or barley (Fig. 7a, Table 4 and S4). Yield reduction in reduced tillage systems, particularly during the transition phase, has been reported but the effect size will be context dependent (Van Den Putte et al. 2010; Pittelkow et al. 2015). Reduced tillage was reported to decrease crop yield by 5.1% on average compared to conventional tillage at a global scale, and crop type was the most important factor affecting the yield response to reduced tillage (Pittelkow et al. 2015). Analysis of five site-years data in three trials in France and Switzerland also showed that the effect of reduced tillage on grain yield depends on soil type, weather and time after conversion (Peigné et al. 2014). The fact that cultivation was only a significant model term for wheat yield in combination with site and other factors (Table 4) illustrates the value of taking a systems approach to long-term experimentation in understanding the likely impact of a management change in different contexts (Pittelkow et al. 2015; Samson et al. 2019). For example, the negative impact of reduced tillage was greater for second wheats and, at Harpenden, for wheat following a grass/clover ley (Fig. 7a). This latter effect was explained by the difficulty of terminating the ley without tillage leading to competition from grass and clover volunteers in the following wheat. This type of finding will therefore be important in establishing and potentially modifying experimental rotations that are better suited for sustainable farming systems.

In some systems of the LSRE, the reduced tillage treatment was in line with the three principles of conservation agriculture—permanent soil cover (e.g., by crops, cover crops or crop residues), minimal soil disturbance (e.g., reduced tillage) and diverse crop species (e.g., via rotation) (FAO 2013). Meta-analyses and reviews have shown that the impacts of conservation agriculture on yield are mixed (Stevenson et al. 2014). Pretty and Bharucha (2014) argued that this might reflect that conservation agriculture is context sensitive, and its outcomes depend on the combination of practices, and differ by crop type. A recent study based on a large database covering eight crops compared the performance between conservation agriculture and conventional tillage (Su et al. 2021). It suggested that conservation agriculture has better performance than systems with reduced tillage alone and a high chance (>50%) to outperform conventional tillage in dryer regions across the world. However, in wetter regions reduced tillage only has a <50% chance of outyielding conventional tillage. In an 18-year study on a vertisol under rainfed Mediterranean conditions, reduced tillage resulted in a grain yield advantage for wheat over conventional tillage when water stress was high but a disadvantage when water stress was low (Amato et al. 2013). There is also evidence that reduced tillage (on its own or combined with other conservation agriculture practices) may result in yield penalties in the short term but reduce with time, ultimately leading to yield benefits (Brouder and Gomez-Macpherson 2014; Pittelkow et al. 2015; MacLaren et al. 2022). This might be the case for the initial stage of the LSRE, and long-term financial support of the LSRE will be vital to provide valuable data to demonstrate accrued benefits to soil function of reduced tillage in the longer term. Derpsch et al. (2010) compared conventional tillage and conservation agriculture over a decadal-scale and found increasing yield under conservation agriculture over time with additional benefits of lower inputs. At the global scale, yields tend to decrease in the first few years after conversion to reduced tillage but can increase after 3–10 years (Pittelkow et al. 2015).

In the present study, spring barley that received additional organic amendment in terms of both cover crops and compost application yielded higher, by 8% on average, than subplots that did not (Fig. 7b). Similar results have been reported earlier (Li et al. 2015). This has likely been due to an excessive N input from organic and inorganic sources, for example, 303 kg N ha^−1^ applied as compost in 2020 in addition to inorganic fertilizer that was applied at the same rate of that in the standard subplots, although most of this will not be available to the crop. This may have also led to low N use efficiency and larger N losses, including leaching. In the early years of the LSRE, crops received typical inputs of inorganic nitrogen fertilizers (Fig. S1). However, in the future, organic and inorganic inputs will be tailored to the requirements of crops in each phase of the system using accrued experience and additional soil and crop measurements. We, therefore, expect the improved nutrient use efficiency of some systems on the LSRE to be reflected in reduced inputs in future years, including optimal use of available organic sources such as compost.

Soil type played an important role in determining beans yield (Table 4). The spring bean yield was constantly higher on the lighter soil at Brooms Barn during the study period (Fig. 7c, Table S4), contradictory to evidence that beans perform poorly on light sandy soils but best on heavier-textured soils (Jensen et al. 2010). However, the site effect was, again, dependent on the system context. The 7-year rotation differs in the sixth phase (preceding spring beans)—linseed at Harpenden and sugar beet at Brooms Barn. This is reflected in the bean yields that are proportionally lower following linseed when compared to the 5-year rotation where the preceding crop (winter wheat) is the same at the two sites.

Besides the above example about the effects of multiple factors on a single variable at the crop level, we also provided an example of analyzing the system performance using calorific yield which can be integrated across different crops. We showed that at the early stage of the LSRE, the common crop rotation strategy with two winter cereals and an oilseed break crop combined with conventional plowing, crop protection and additional organic input tended to produce the highest calories per unit area per year, and all the systems with reduced tillage tended to occupy the bottom half of the ranking of calorific yield (Fig. 6). Except for calorific yield, other unifying metrics, such as the use of water, nutrient, labor, energy and capital, protein and economic yield, environmental footprint such as GHG emissions, can also be calculated, allowing for system level evaluation. Furthermore, with more multi-dimensional data accrued in the longer term, trade-offs and synergies between different components of the system can be analyzed using methods such as radar charts (Davis et al. 2012; Gathala et al. 2013; Colnenne-David and Doré 2015) and structural equation models (Garland et al. 2021). The primary intention of the LSRE is not to provide recommendation to farmers or policymakers the optimal system. Rather, the multi-dimensional data generated in the LSRE will be valuable to answering questions like, which is the most profitable system with the least financial or production risk, and lowest environmental footprint in the long run.

## Conclusions

We documented in the present paper the principles underpinning the design of a new generation of field experiments exemplified by the new Large Scale Rotation Experiment at Rothamsted Research in the UK. It was developed using an inter-disciplinary approach based on experiences from some of the world’s longest-running field experiments in order to address the increasing societal demands for twenty-first century agriculture to not only provide food, feed and fiber but also public goods and services. This new type of experiment incorporates multiple management interventions pertinent to many alternative strategies for sustainable/regenerative agriculture with some flexibility to incorporate novel management or test new hypotheses. The LSRE was designed to be statistically robust but allowing for practical constraints and dynamic management changes, and with modern data analytic techniques in mind. The analysis of the yields of wheat, barley and beans is a first validation of the need for an experiment of this type. The significant interaction effects between the management factors and between management and site highlighted the importance of the management and environmental context in understanding the effect of an intervention such as reduced tillage and the value of establishing the experiment at more than one site.

In the future, the LSRE aims to evaluate the importance of a long-term commitment to accruing multi-dimensional data at the systems level to provide the evidence base for alternative pathways to sustainable agriculture. The multi-dimensional data accrued over time will have the potential for system-level evaluation of the synergies and trade-offs of multiple factors on emerging variables of interests as well as traditional productivity parameters using not only traditional analytical frameworks but also novel statistical approaches. The resulting new understanding of system behavior and models for predicting the system response of different outcomes to multiple drivers will be important resources for farmers and policymakers in guiding decisions on how to modify existing systems to reconcile multiple objectives.

### Supplementary Information

Below is the link to the electronic supplementary material.

Supplementary file1 (DOCX 96 kb)

## Data Availability

The data used in this study are available upon request in Electronic Rothamsted Archive, Rothamsted Research.

## Code availability

The R code used to fit linear mixed models to the yield data is available upon request.
