# Supplementary material for: A new Rothamsted long-term field experiment for the twenty-first century: principles and practice
Source: Agron Sustain Dev. 2023 Aug 24;43(5):60. doi: 10.1007/s13593-023-00914-8 (PMC10449994; doi:10.1007/s13593-023-00914-8)
Supplement: Supplementary file 1 — Supplementary file1 (DOCX 96 kb) [file 13593_2023_914_MOESM1_ESM.docx]

Journal: *Agronomy for Sustainable Development*

*Supplementary Materials*

**A new Rothamsted long-term field experiment for the 21^st^ Century: principles and practice**

XIAOXI LI^†^, JONATHAN STORKEY^*^, ANDREW MEAD, IAN SHIELD, IAN CLARK, RICHARD OSTLER, BETH ROBERTS, ACHIM DOBERMANN

*Rothamsted Research, West Common, Harpenden, Hertfordshire, AL5 2JQ, UK*

*^*^Corresponding author*: Jonathan Storkey, Protecting Crops and the Environment, Rothamsted Research, West Common, Harpenden, Hertfordshire, AL5 2JQ, UK. Tel: (+44) 1582 938550; E-mail: [jonathan.storkey@rothamsted.ac.uk](mailto:jonathan.storkey@rothamsted.ac.uk)

^†^*Present address:* CSIRO Agriculture and Food, Canberra, ACT, 2601, Australia

**Table S1** Unique systems subject to combinations of the four management factors in the LSRE.

| System | Rotation | Cultivation | Crop protection | Nutrition |
| --- | --- | --- | --- | --- |
| R3_CT_Con_Amd | 3-year | Conventional tillage | Conventional | Amended |
| R3_CT_Con_Std | 3-year | Conventional tillage | Conventional | Standard |
| R3_CT_SCP_Amd | 3-year | Conventional tillage | Smart crop protection | Amended |
| R3_CT_SCP_Std | 3-year | Conventional tillage | Smart crop protection | Standard |
| R3_RT_Con_Amd | 3-year | Reduced tillage | Conventional | Amended |
| R3_RT_Con_Std | 3-year | Reduced tillage | Conventional | Standard |
| R3_RT_SCP_Amd | 3-year | Reduced tillage | Smart crop protection | Amended |
| R3_RT_SCP_Std | 3-year | Reduced tillage | Smart crop protection | Standard |
| R5_CT_Con_Amd | 5-year | Conventional tillage | Conventional | Amended |
| R5_CT_Con_Std | 5-year | Conventional tillage | Conventional | Standard |
| R5_CT_SCP_Amd | 5-year | Conventional tillage | Smart crop protection | Amended |
| R5_CT_SCP_Std | 5-year | Conventional tillage | Smart crop protection | Standard |
| R5_RT_Con_Amd | 5-year | Reduced tillage | Conventional | Amended |
| R5_RT_Con_Std | 5-year | Reduced tillage | Conventional | Standard |
| R5_RT_SCP_Amd | 5-year | Reduced tillage | Smart crop protection | Amended |
| R5_RT_SCP_Std | 5-year | Reduced tillage | Smart crop protection | Standard |
| R7_CT_Con_Amd | 7-year | Conventional tillage | Conventional | Amended |
| R7_CT_Con_Std | 7-year | Conventional tillage | Conventional | Standard |
| R7_CT_SCP_Amd | 7-year | Conventional tillage | Smart crop protection | Amended |
| R7_CT_SCP_Std | 7-year | Conventional tillage | Smart crop protection | Standard |
| R7_RT_Con_Amd | 7-year | Reduced tillage | Conventional | Amended |
| R7_RT_Con_Std | 7-year | Reduced tillage | Conventional | Standard |
| R7_RT_SCP_Amd | 7-year | Reduced tillage | Smart crop protection | Amended |
| R7_RT_SCP_Std | 7-year | Reduced tillage | Smart crop protection | Standard |

**Table S2** Monthly total and annual precipitation, and monthly and annual mean air temperature at Brooms Barn during October 2017-September 2021. “LT mean” is the long-term mean value at the site during 1991-2020.

|  | Precipitation (mm) | | | | | Temperature (℃) | | | | | |  |
| --- | --- | --- | --- | --- | --- | --- | --- | --- | --- | --- | --- | --- |
|  | 2017-18 | 2018-19 | 2019-20 | 2020-21 | LT mean | | 2017-18 | 2018-19 | 2019-20 | 2020-21 | LT mean | |
| Oct | 19.8 | 60.2 | 84.2 | 113.0 | 62.5 | | 12.4 | 11.6 | 10.8 | 11.0 | 11.3 | |
| Nov | 39.2 | 48.4 | 76.2 | 48.5 | 62.0 | | 6.5 | 8.3 | 6.3 | 8.7 | 7.4 | |
| Dec | 86.0 | 59.0 | 86.4 | 117.6 | 57.7 | | 4.0 | 6.9 | 6.0 | 4.6 | 4.8 | |
| Jan | 66.3 | 25.8 | 53.6 | 81.1 | 52.2 | | 4.8 | 3.6 | 6.4 | 2.8 | 4.4 | |
| Feb | 41.4 | 30.6 | 90.8 | 38.3 | 43.4 | | 2.4 | 7.1 | 6.6 | 5.0 | 4.7 | |
| Mar | 78.7 | 47.4 | 27.6 | 39.8 | 41.1 | | 4.8 | 8.4 | 6.7 | 7.1 | 6.8 | |
| Apr | 68.9 | 12.2 | 34.2 | 2.2 | 41.0 | | 10.3 | 8.7 | 10.9 | 6.3 | 9.2 | |
| May | 27.8 | 62.6 | 4.6 | 83.9 | 50.2 | | 14.0 | 11.9 | 13.3 | 10.6 | 12.3 | |
| Jun | 1.6 | 99.2 | 86.4 | 89.2 | 58.5 | | 17.0 | 15.5 | 15.9 | 16.4 | 15.2 | |
| Jul | 18.6 | 37.8 | 55.2 | 45.0 | 56.8 | | 20.7 | 18.9 | 17.1 | 18.0 | 17.6 | |
| Aug | 69.4 | 40.6 | 82.8 | 38.0 | 67.1 | | 18.4 | 18.7 | 19.2 | 16.5 | 17.5 | |
| Sep | 31.2 | 65.0 | 49.8 | 72.2 | 51.1 | | 15.0 | 15.3 | 15.2 | 16.9 | 14.9 | |
| *Year* | *548.9* | *588.8* | *731.8* | *768.8* | *643.6* | | *10.9* | *11.2* | *11.2* | *10.3* | *10.5* | |

**Table S3** Monthly total and annual precipitation, and monthly and annual mean air temperature at Harpenden during October 2017-September 2021. “LT mean” is the long-term mean value at the site during 1991-2020.

|  | Precipitation (mm) | |  |  |  | Temperature (℃) | |  |  |  |
| --- | --- | --- | --- | --- | --- | --- | --- | --- | --- | --- |
|  | 2017-18 | 2018-19 | 2019-20 | 2020-21 | LT mean | 2017-18 | 2018-19 | 2019-20 | 2020-21 | LT mean |
| Oct | 31.1 | 71.0 | 109.6 | 198.4 | 81.3 | 12.3 | 10.9 | 10.5 | 10.9 | 10.9 |
| Nov | 53.2 | 63.8 | 91.0 | 57.4 | 81.2 | 6.6 | 7.9 | 6.1 | 8.4 | 7.2 |
| Dec | 110.7 | 75.0 | 111.6 | 98.4 | 75.6 | 4.5 | 6.5 | 5.8 | 4.9 | 4.7 |
| Jan | 76.1 | 34.8 | 79.8 | 113.6 | 74.1 | 5.2 | 3.4 | 6.4 | 3.1 | 4.3 |
| Feb | 48.5 | 43.2 | 127.0 | 45.8 | 56.9 | 2.3 | 6.2 | 6.5 | 5.2 | 4.5 |
| Mar | 78.3 | 60.4 | 43.8 | 27.6 | 47.1 | 4.9 | 7.9 | 6.7 | 7.1 | 6.6 |
| Apr | 75.0 | 13.2 | 51.4 | 1.6 | 54.0 | 10.3 | 8.8 | 10.7 | 6.4 | 8.9 |
| May | 61.9 | 42.8 | 3.2 | 94.4 | 53.3 | 13.3 | 11.3 | 12.9 | 10.5 | 11.9 |
| Jun | 3.5 | 70.8 | 87.4 | 77.6 | 54.8 | 16.2 | 14.7 | 15.7 | 16.1 | 14.9 |
| Jul | 15.1 | 45.0 | 81.2 | 51.0 | 55.5 | 19.9 | 18.1 | 16.7 | 17.8 | 17.2 |
| Aug | 64.0 | 45.2 | 172.0 | 37.0 | 72.0 | 17.4 | 17.8 | 18.6 | 16.1 | 17.0 |
| Sep | 51.0 | 75.0 | 32.8 | 54.1 | 57.7 | 14.1 | 14.9 | 14.7 | 16.1 | 14.4 |
| *Year* | *668.4* | *640.2* | *990.8* | *856.9* | *763.5* | *10.6* | *10.7* | *10.9* | *10.2* | *10.2* |

**Table S4** Summary statistics of yield (Mg ha^-1^) of the three grain crops grouped by site×cultivation for 2020 and 2021. The mean value was calculated over the crop protection and nutrition factors based on the raw data. sd: standard deviation, n: number of observations in each group.

| Crop | Site | Cultivation | 2020 |  |  | 2021 |  |  |
| --- | --- | --- | --- | --- | --- | --- | --- | --- |
|  |  |  | Mean | sd | n | Mean | sd | n |
| Barley | Brooms Barn | Conventional tillage | 5.89 | 0.61 | 8 | 6.57 | 0.41 | 8 |
|  |  | Reduced tillage | 5.78 | 0.71 | 8 | 5.80 | 0.73 | 8 |
|  | Harpenden | Conventional tillage | 6.75 | 0.28 | 8 | 4.53 | 1.14 | 8 |
|  |  | Reduced tillage | 4.68 | 0.88 | 8 | 2.17 | 0.62 | 8 |
| Beans | Brooms Barn | Conventional tillage | 4.28 | 0.42 | 8 | 5.67 | 0.33 | 8 |
|  |  | Reduced tillage | 3.17 | 0.43 | 8 | 5.23 | 0.83 | 8 |
|  | Harpenden | Conventional tillage | 3.09 | 1.05 | 8 | 2.60 | 0.69 | 8 |
|  |  | Reduced tillage | 1.52 | 1.06 | 8 | 2.61 | 0.71 | 8 |
| Wheat | Brooms Barn | Conventional tillage | 7.10 | 1.46 | 24 | 9.49 | 1.21 | 24 |
|  |  | Reduced tillage | 5.90 | 1.42 | 24 | 9.13 | 0.96 | 24 |
|  | Harpenden | Conventional tillage | 6.70 | 0.76 | 24 | 7.14 | 1.47 | 24 |
|  |  | Reduced tillage | 6.11 | 1.64 | 24 | 6.52 | 2.32 | 20 |

**Fig. S1.** Typical sowing and harvest dates for crops grown on the LSRE with fertiliser events indicated with vertical lines on calendar for each crop and total typical inorganic nitrogen inputs reported in final column.

***Supplementary material – Implementation of treatments and management of the LSRE***

*Crop management*

The experiment at Brooms Barn was established in 2018 (i.e., winter crops were sown in autumn 2017) while in 2019 at Harpenden. This was intended to avoid collision of intensive samplings planned every third year (See below). The space between adjacent plots was 2 and 12 m along the rows and columns of plots, respectively (Fig. 3). Field operations were implemented along the columns of plots. A preparatory crop of winter oats was sown to all plots at both sites prior to the establishment of the experiment. Information on the sowing and harvest dates of each crop and typical rates and timings of nitrogen fertilisation is provided in Fig. S1. Crops were established in the conventional tillage treatment at both sites using an Accord 3m combination drill and in the reduced tillage treatment using a Weaving 3m GD drill at Brooms Barn and a Simtech 3m t-Sem 300 at Harpenden. The grass/clover ley was cut for hay once or twice a year during June-August. Crop harvestable parts (i.e., grain, beet) and hay are routinely analysed every year for contents of nitrogen, carbon and other elements, oilseed rape for oil content, and sugar beet for sugar content. Cover crops are sampled in February each year to measure the biomass and nutrient content.

*Implementation of treatments*

There were some alterations to the original treatments during the first four years of the LSRE (2018-2021). Compost was not applied until the second season at both sites, i.e., starting from 2019 at Brooms Barn and 2020 at Harpenden. Cover crops were sown to both subplots of relevant phases (Fig. 2) until 2020, after this, they have only been grown on the amended side. Grass/clover ley (phase C3) was undersown to the preceding spring barley (C2), not tilled and not applied with crop protection treatments during its growth. The smart crop protection treatment was not applied in 2018 but started in 2019 at both sites, and it was only possible to apply the smart crop protection principles to winter crops, not spring crops.

It is a challenge to deal with crop failure both in terms of crop management and statistical analysis. Soybean (C7, four main-plots per site) did not reach maturity at either site in 2018 and 2019, and was discontinued and substituted with spring field beans from 2020. Failed soybean and linseed crops were not replaced with any alternative. There were always some main-plots with failed winter OSR at both sites, e.g., all OSR at Harpenden and that under conventional tillage at Brooms Barn in 2021 failed, mainly because of poor establishment in relation to either poor weather or pest pressure. Plots with failed OSR were resown to mustard in the following spring as a cover crop, and failed winter wheat replaced with spring wheat, e.g., around half of the winter wheat main-plots at Harpenden in both 2020 and 2021. Both winter and spring wheat yield data were included in the statistical analysis and not differentiated in this paper but the need to resow a main-plot was effectively included as a treatment effect reflected in yields.

Compost was applied at a constant rate of 30 t ha^-1^ (wet-wt basis). The total N content (dry-wt basis) was 1.3 and 1.6%, and thus the total N applied as compost was 241 and 303 kg N ha^-1^ in 2019 and 2020, respectively.

*Residue Management*

Upon establishing the experiment, it was decided that straw would be left on all experimental plots and incorporated into the soil either through inversion tillage in the conventional tillage main-plots or left to incorporate through natural processes on the reduced tillage main-plots. However, from 2020 it was decided that straw would be removed in the 3-year rotation from the standard subplots only as an addition to the nutrition treatment, with the straw being left to incorporate on the amended side of the subplot. This decision was based upon the principal of the 3-year rotation system being concerned with maximising profits not environmental gains, such as increasing SOC by incorporating straw. Straw will always be incorporated in the 7-year rotation as this system prioritises the environment and natural capital over short-term economics. The 5-year rotation remains a middle ground, based on agronomic best practice, so discussions about removing the straw intermittently (e.g., once in four years) when straw price is high will continue.

*Baseline measurements*

A series of baseline measurements were taken at the subplot level in August before the drilling of autumn sown crops in the first season at both sites: 1) Ten 0-23 cm soil cores taken from each subplot using a 3-cm diameter auger and bulked; soil analysed for total nitrogen and carbon (using a LECO TruMac Combustion Analyser), major and trace elements (using a Perkin Elmer NexION 300X ICP-MS analyser), Olsen P, bulk density and pH (H_2_O, 1:2.5), 2) Single 10-cm diameter deep core taken at the centre of each main-plot and analysed for total carbon and nitrogen, major and trace elements, Olsen P, bulk density and pH at two depths: 23-60 and 60-100 cm, 3) Ten 0-23 cm soil cores taken from 36 subplots sampled at random across the experimental field using a 3-cm diameter auger and bulked to analyse soil microbiology. A 50 g homogenised sample per subplot was frozen in the field using liquid nitrogen and next generation amplicon sequencing of bacterial 16S rRNA genes and fungal Internal Transcribed Spacer (ITS) regions analysed to derive baseline taxonomic diversity indices, 4) weed seedbank samples; three sampling locations georeferenced in each sub plot along a diagonal transect, soil sampled to a depth of approximately 12 cm using a trowel at each sample point and bulked at the subplot level. Soil was sieved to remove stones and placed in seed trays to a depth of 4 cm in an unheated, unlit glasshouse. The trays were kept well-watered and seedlings identified and removed at regular intervals between September and April, 5) penetrometer measurements (to 1 m) taken in each main-plot.

In addition to these routine measurements (repeated every third year), since the establishment of the LSRE a range of additional variables have also been measured on sub-sets of main-plots over different time periods to address specific research questions. These include data on soil physical properties, biodiversity (earthworms and beneficial arthropods), GHGs and pest and disease pressure (Fig. 5).

***Supplementary material – Statistical consideration for the design of the LSRE***

The LSRE was designed with two main contrasting approaches to data analysis in mind. The first uses the factorial treatment structure and allows the assessment of the main effects and low-order interactions within an ANOVA framework. Such assessment can be done either at the test crop level (e.g., on grain yield) or the rotation level (e.g., calorific yield). Where comparable measurements can be made across all plots simultaneously, such an analysis can assess the impacts of all four management interventions (rotation, cultivation, nutrition and crop protection), considering all main effects and two-, three- and four-factor interactions using the multiple phases within each rotation to provide replication. Where simultaneous comparable measurements need to be calculated by integrating responses across the different phases of each rotation within the combinations of the other factors (i.e. aggregating responses across multiple main-plots or subplots), the variation due to main effects and low-order interactions can only be assessed relative to the variation due to higher-order interactions, with three- and four-factor interactions being assigned to the residual term (a commonly used approach where there is no true replication for a factorial treatment structure). Once data are available across several years for each main-plot or subplot, comparable measurements can be obtained by integrating responses over time (rather than space), so that the main-plots (and subplots) within a rotation again provide some true replication of the effects of the management interventions, the only challenge here being the different lengths of the rotations, so that different numbers of complete cycles will have been observed for each rotation within a particular duration of study. An alternative approach is the so-called “space-for-time” substitution using the “fully-phased” nature of the design. We can consider the years as providing the replication, integrating the responses across the different phases of each rotation within each year, for each combination of the other factors. For those crops that are common across rotations, and even repeated within a rotation, the fully phased nature of the design means that multiple main-plots of each crop are present in every year, so that it is also possible to statistically compare metrics (such as yield) at the level of these ‘test crops’ to quantify the impacts of the cropping system context. In this case, year can be included as an additional source of error in a linear mixed model (as in this paper) or expressed in terms of meteorological summaries as additional explanatory terms where the impact of seasonality is of interest.

Secondly, the co-variance of state and outcome variables in response to the treatments can be analysed using multivariate analysis (such as Principal Components Analysis or Redundancy Analysis) or, to draw conclusions on the directionality and causality of relationships, e.g., using regression modelling or Structural Equation Modelling. In this case, the treatments are not the focus of the analysis but, rather, a means of achieving gradients of related variables and the experiment is used to gain insights into the behaviour of the system in terms of the relationships between state variables and outcomes.

The following are the statistical models used in this paper for the global analysis on all yield data of the three crops in 2020-2021 (eq. 1), subsets of wheat (eq. 2), barley (eq. 3) and beans (eq. 4), respectively,

*Yield = µ + Ct + Nu + Pr + St + Cr.Rt + Ct×Nu + Ct×Pr + Ct×St + Nu×Pr + Nu×St + Pr×St + Ct×Cr.Rt + Nu×Cr.Rt + Pr×Cr.Rt + St×Cr.Rt + Ct×Nu×Pr+ Ct×Nu×St + Ct×Pr×St + Nu×Pr×St + Ct×Nu×Cr.Rt + Ct×Pr×Cr.Rt + Ct×St×Cr.Rt + Nu×Pr×Cr.Rt + Nu×St×Cr.Rt + Pr×St×Cr.Rt + Ct×Nu×Pr×St + ε*  (1)

*Yield = µ + Ct + Nu + Pr + St + Pre.Rt + Ct×Nu + Ct×Pr + Ct×St + Nu×Pr + Nu×St + Pr×St + Ct×Pre.Rt + Nu×Pre.Rt + Pr×Pre.Rt + St×Pre.Rt + Ct×Nu×Pr+ Ct×Nu×St + Ct×Pr×St + Nu×Pr×St + Ct×Nu×Pre.Rt + Ct×Pr×Pre.Rt + Ct×St×Pre.Rt + Nu×Pr×Pre.Rt + Nu×St×Pre.Rt + Pr×St×Pre.Rt + Ct×Nu×Pr×St + ε*  (2)

*Yield = µ +Rt + Ct + Nu + Pr + St + Rt×Ct + Rt×Nu + Rt×Pr + Rt×St +Ct×Nu + Ct×Pr + Ct×St + Nu×Pr + Nu×St + Pr×St + Rt×Ct×Nu + Rt×Ct×Pr + Rt×Ct×St + Rt×Nu×Pr + Rt×Nu×St + Rt×Pr×St + Ct×Nu×Pr+ Ct×Nu×St + Ct×Pr×St + Nu×Pr×St + Rt×Ct×Nu×Pr+ Rt×Ct×Nu×St + Rt×Ct×Pr×St + Rt×Nu×Pr×St + Ct×Nu×Pr×St + ε*  (3)

*Yield = µ + Ct + Nu + Pr + St.Rt + Ct×Nu + Ct×Pr + Nu×Pr + Ct×Nu×Pr+ Ct×St.Rt + Nu×St.Rt + Pr×St.Rt + Ct×Nu×St.Rt + Ct×Pr×St.Rt + Nu×Pr×St.Rt + ε*  (4)

where *µ* is the overall mean yield, Ct: Cultivation (reduced tillage vs. inversion tillage), Nu: Nutrition (standard fertilisation vs. additional organic amendment), Pr: Crop protection (conventional crop protection vs. smart crop protection), St: site, Cr: crop (wheat, barley and beans), Rt: Crop rotation (3-, 5- or 7-year), Pre: crop preceding wheat (pre-crop), and *ε* error term. *Cr.Rt,* *Pre.Rt* and *St.Rt* were created to account for the respective factors due to their partial crossed nature in individual models (See Materials and methods section).
